# Sub-MIC levels of bedaquiline and clofazimine can select *Mycobacterium tuberculosis* mutants with increased MIC

**DOI:** 10.1128/aac.01275-23

**Published:** 2024-03-12

**Authors:** Cristina Villellas, Frederik Stevenaert, Bart Remmerie, Koen Andries

**Affiliations:** 1 Janssen Research and Development, Beerse, Belgium; St George's, University of London, London, United Kingdom

**Keywords:** bedaquiline, tuberculosis, drug resistance, clofazimine, *Rv0678*

## Abstract

Multidrug-resistant tuberculosis (MDR-TB) patients not cured at the time of stopping treatment are exposed to Minimum Inhibitory Concentration (MIC) and sub-MIC levels for many months after discontinuing bedaquiline (BDQ) or clofazimine (CFZ) treatment. *In vitro* cultures treated with BDQ and CFZ sub-MIC concentrations clearly showed enrichment in the *Rv0678* mutant population, demonstrating that pre-existing *Rv0678* mutants can be selected by sub-MIC concentrations of BDQ and CFZ if not protected by an alternative MDR-TB treatment.

## INTRODUCTION

Bedaquiline (BDQ)-containing regimens have recently become standard of care for all patients with rifampin-resistant tuberculosis (TB) (1). Resistance to BDQ in clinical TB infections is almost entirely attributable to mutations in *Rv0678* (*mmpR5*), which may also confer resistance to clofazimine (CFZ) (2–4). Pre-existing *Rv0678* mutants can easily be selected *in vitro* when TB cultures with a size exceeding the spontaneous mutation rate [4.7 × 10^−7^-8.9 x10^−9^ mutations per cell per division (5)] are exposed to BDQ or CFZ concentrations exceeding their wild-type MIC.

However, resistant mutants can also be selected at wild-type sub-MIC concentrations, if the benefit of the resistant mutation in the presence of the antibiotic outweighs their fitness cost, as has been shown for *Escherichia coli* and *Salmonella enterica* (6). This is relevant for BDQ and CFZ, as both drugs are very lipophilic, accumulate in fatty tissues and macrophages, and are slowly released from tissues once treatment has been discontinued, with a terminal half-life in plasma of 164 days for BDQ (7) and 34.2 days [estimated, (8)] for CFZ.

The washout pharmacokinetics (PK) profiles in multidrug-resistant tuberculosis (MDR-TB) patients after 2 and 6 months of BDQ treatment were reported previously (7, 9). In both studies, plasma concentrations of BDQ were above the MIC for many months after stopping BDQ and were still quantifiable in almost all patients at study completion, 96 weeks after finishing treatment.

In earlier MDR-TB regimens, BDQ was administered for 6 months in a regimen with a total duration of 18 months (1). In this scenario, the tail of gradually decreasing plasma levels of BDQ between month 6 and month 18 was protected by the presence of companion drugs. In shorter regimens for treatment of MDR TB as nowadays recommended by the World Health Organization [e.g., Bedaquiline, Pretomanid and Linezolid (BPaL)], this is no longer the case, and there may be an increased risk for selecting pre-existing resistant variants if the patient is not culture negative at the end of treatment. Our aim was to investigate *in vitro* whether BDQ or CFZ could select for *Rv0678* mutations when plasma concentrations drop below their wild-type MIC.

To assess the minimum drug concentration that favored selection of a *Rv0678* mutant vs wild type, we compared the relative growth *in vitro* of the *Mycobacterium tuberculosis* wild-type strain H37Rv (wild type, wt) and CV37 (mutant, mt), an H37Rv derivative carrying a big insertion (insertion sequence *IS*6110) disrupting the efflux pump regulator gene *Rv0678* at nucleotide position 104. The MICs of BDQ and CFZ are 0.06 and 0.25 mg/L for the wild-type H37Rv and 0.24 and 4 mg/L for strain CV37, respectively. Green fluorescent protein (GFP) and DsRed2-marked strains were generated for the wild-type and the *Rv0678* mutant (Table S1). The competition experiments were performed as adapted from Gullberg et al. (6). Cultures were grown in 7H9/10% OADC/0.05% Tween 80 broth to the logarithmic phase and diluted to ∼5 × 10^4^ CFU/mL. Two independent flasks were inoculated with (i) wt-DsRed2 and mt-GFP and (ii) wt-GFP and mt-DsRed2, aiming to have similar counts of wt and mt bacteria. Each of the mixed cultures were divided in 5 mL aliquots and compounds were added in a twofold serial dilution (range: 0.06–0.0038 mg/L). Both BDQ and CFZ were done in duplicate and three flasks remained antibiotic-free as control. After incubation for 1 week at 37°C, 500 µL aliquots from all cultures were inactivated in 4% paraformaldehyde and analyzed using a fluorescence-activated cell sorter in FACS Fortessa (method in Supplementary information). The percentage of GFP-containing and DsRed2-containing bacilli was counted in each sample and the ratio of CV37/H37Rv (mt/wt) was calculated for each condition. On the same day, every culture was passaged at a dilution of 1:1000 to a new flask containing the same concentration of compound. These steps of sampling and passaging were repeated every week for 4 weeks.

**Fig 1:**
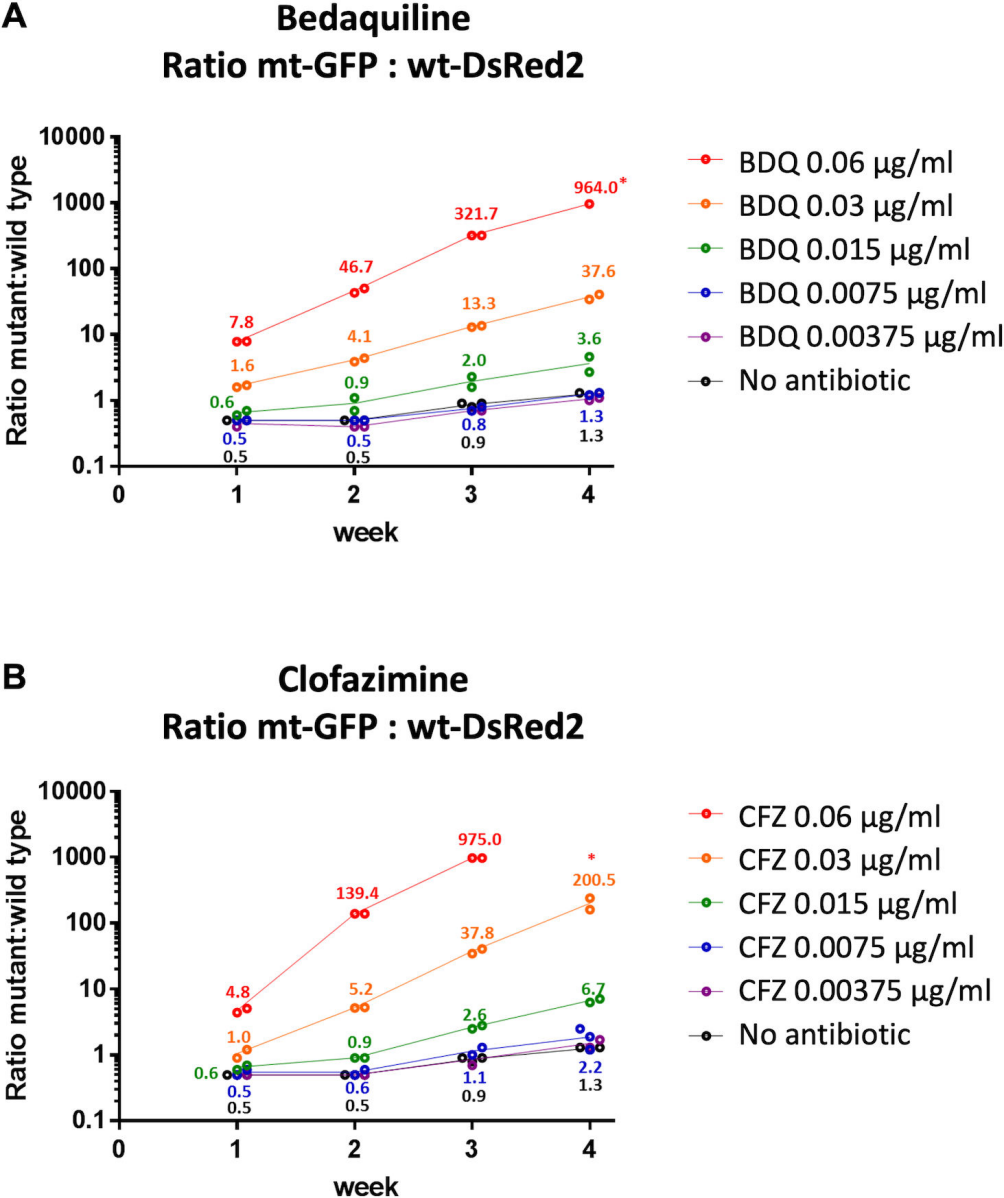
Ratios of mutant vs wild type for all the concentrations of BDQ and CFZ tested, over 4 weeks. The *M. tuberculosis* BDQ-resistant mutant, CV37 (mt-GFP), and the wild-type strain, H37Rv (wt-DsRed2), were pooled and passaged weekly for a total of 4 weeks, in the presence of subinhibitory concentrations of BDQ (**A**) and CFZ (**B**). The alternative pair (mt-DsRed2 vs wt-GFP) is shown in Fig. S2. Fluorescent-marked bacteria were counted by flow cytometry, and the ratios of the percentage of mt and wt were calculated per point. Each condition was performed in duplicate, except for the cultures containing no antibiotic, that was done in triplicate. Individual values are plotted, and the average is indicated. *For some points of BDQ and CFZ at the highest concentrations, there was no wt population detected (<0.1%) at week 4, so the ratios could not be calculated.

Cultures treated with BDQ and CFZ at wild-type MIC or sub-MIC concentrations over 4 weeks clearly showed enrichment in the *Rv0678* mutant population (Fig. 1; Table S2; Fig. S2). Our experiments demonstrate that *Rv0678* mutants can be selected by wild-type MIC and sub-MIC concentrations of BDQ and CFZ (down to 0.015 mg/L for BDQ and 0.015 mg/L for CFZ).

There are limitations of this study. The concentrations selected for in this *in vitro* study are somewhat arbitrary and may not reflect actual drug levels in tissues. One cannot estimate the time window (after stopping treatment) in which selection of resistance mutants is a risk, as it may be different in different tissues. Nevertheless, all tissues will be exposed to sub-MIC levels at some point in time after stopping treatment. The second limitation is the artificial setup, in which a population of ~50% wild-type bacteria is competing with ~50% *Rv0678* mutants. In patients, a minority of pre-existing *Rv0678* mutants [mean 1 in 1.8 × 10^7^ bacteria (5)] is competing with a majority of wild-type bacteria.

The long terminal half-lives of both BDQ and CFZ imply that patients not cured at the time of stopping treatment will be exposed to MIC and sub-MIC levels for many months after all drugs are stopped and may select *Rv0678* mutations. Although this is an alarming observation, emerging data suggest that *Rv0678* mutations certainly do not emerge in each and every patient in this situation. For CFZ, no *Rv0678* mutants were found in seven patients that returned for treatment in study C209 after failing on a CFZ-based regimen (2). For BDQ, emergence of *Rv0678* mutations was observed in only a small proportion of patients that previously failed a BDQ-based regimen (3, 4, 10), although in a recent retrospective study, a much higher frequency was found, likely due to the extensive resistance of the isolates (11).

To preserve the value of BDQ, more studies are needed to understand why some patients develop resistance and others do not. Whether *Rv0678* mutants do emerge may depend on the bacterial load at the time of stopping MDR-TB treatment and the presence of pre-existing *Rv0678* mutants at that time. For the reasons mentioned above, patients lost to follow-up early in their treatment course, before the bacterial burden is reduced to critical levels (below the threshold for spontaneous mutations), may have a significant risk of developing resistance to both BDQ and CFZ. Patients in which BDQ or CFZ is discontinued because of adverse events must be treated with an alternative MDR-TB treatment regimen to prevent the selection of *Rv0678* mutants.

## SUPPLEMENTAL MATERIAL

Supplemental methods, tables and figures aac.01275-23-s0001.docx Supplemental methods (Flow Cytometric analysis to determine ratios of differentially labelled mutant vs wild type strains), Table S1 (Strains used in this study), Table S2 (Mean values and individual replicates of the ratios of mutant vs wild type for all the concentrations of BDQ and CFZ tested, over 4 weeks), Fig. S1 (Flow cytometric analysis of transformed TB cultures), Fig. S2 (Ratios of mutant vs wild type for all the concentrations of BDQ and CFZ tested, over 4 weeks), and Fig. S3 (Fitness of GFP vs DsRed2 strains).

ASM does not own the copyrights to Supplemental Material that may be linked to, or accessed through, an article. The authors have granted ASM a non-exclusive, world-wide license to publish the Supplemental Material files. Please contact the corresponding author directly for reuse.
