# Supplementary material for: Sub-MIC levels of bedaquiline and clofazimine can select Mycobacterium tuberculosis mutants with increased MIC
Source: Antimicrob Agents Chemother. 2024 Mar 12;68(4):e01275-23. doi: 10.1128/aac.01275-23 (PMC10989023; doi:10.1128/aac.01275-23)
Supplement: Supplemental methods, tables and figures — Supplemental methods (Flow Cytometric analysis to determine ratios of differentially labelled mutant vs wild type strains), Table S1 (Strains used in this study), Table S2 (Mean values and individual replicates of the ratios of mutant vs wild type for all the concentrations of BDQ and CFZ tested, over 4 weeks), Fig. S1 (Flow cytometric analysis of transformed TB cultures), Fig. S2 (Ratios of mutant vs wild type for all the concentrations of BDQ and CFZ tested, over 4 weeks), and Fig. S3 (Fitness of GFP vs DsRed2 strains). [file aac.01275-23-s0001.docx]

**Supplemental material**

**Supplemental methods**

**Flow Cytometric analysis to determine ratios of differentially labelled mutant vs wild type strains.**

After incubation for 1 week at 37°C, 500 µL aliquots from all cultures were washed with 200µL PBS, inactivated for 90 min at 4 °C in 400µL 4% paraformaldehyde, and finally resuspended in 200µL PBS. Serial decimal dilutions of inactivated TB cultures were analyzed by flow cytometry using a LSR Fortessa (BD Biosciences, San Jose, CA) running BD FACSDiva software (version 6.0). At least 10000 bacteria were acquired for each condition. The FCS files were analyzed using Microsoft Office Excel. To allow correct gating of the TB bacteria FSC and SSC channels were log transformed. The DsRed and GFP fluorescent proteins were excited using a yellow green (561nm) and blue laser (488nm), respectively. The ratios of the percentages in the DsRed single positive and GFP single positive gate were determined for each condition.


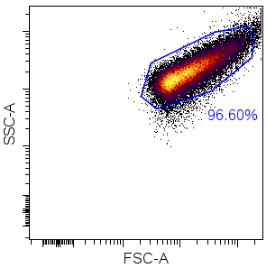

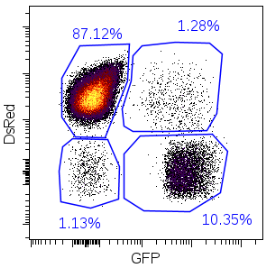


**Fig S1. Flow cytometric analysis of transformed TB cultures:** Representative dot plots showing the gating strategy to allow determination of the ratios of the percentages of each TB strain. Both the wild type and mutant TB strain were generated to express the DsRed and GFP fluorescent protein to determine impact of expression of the fluorescent protein on bacterial fitness. Left dot plot show scatter properties of TB bacteria. Left dot plot shows the expression of the fluorescent proteins on the gated TB population. The percentage of bacteria in the single positive DsRed2 and GFP gates were used in the downstream analysis.

| **Name** | **Strain** | **Plasmid** | **Notes** | **Reference** |
| --- | --- | --- | --- | --- |
| wt-DsRed2 | H37Rv | pND239 (Hyg-R) (1) | BDQ susceptible strain, producing DsRed2 (λem 575nm, λex 632nm) | This study |
| wt-GFP | H37Rv | pND235 (Km-R) (2) | BDQ susceptible strain, producing GFP (λem 475nm, λex 525nm) | This study |
| mt-DsRed2 | CV37 | pND239 (Hyg-R) (1) | BDQ-resistant strain, mutation *Rv0678* *IS*6110 nt 104. It produces DsRed2 (λem 575nm, λex 632nm) | This study |
| mt-GFP | CV37 | pND235 (Km-R) (2) | BDQ-resistant strain, mutation *Rv0678* *IS*6110 nt 104. It produces GFP (λem 475nm, λex 525nm) | This study |

**Table S1**. Strains used in this study. Hyg-R, hygromycin-resistant, Km-R, kanamycin resistant.

References:

1. Manina G, Dhar N, McKinney JD. 2015. Stress and host immunity amplify Mycobacterium tuberculosis phenotypic heterogeneity and induce nongrowing metabolically active forms. Cell Host Microbe 17:32-46.
2. Wakamoto Y, Dhar N, Chait R, Schneider K, Signorino-Gelo F, Leibler S, McKinney JD. 2013. Dynamic persistence of antibiotic-stressed mycobacteria. Science 339:91-5.

|  |  | *Ratio mutant vs wild type (****mean*** *[replicates])* | | | |  |
| --- | --- | --- | --- | --- | --- | --- |
| **Strains** | **Antibiotic** | **week 1** | **week 2** | **week 3** | **week 4** | |
| **mt-GFP:**  **wt-DsRed2** | **BDQ 0.06µg/ml** | **7.8** [7.8, 7.9] | **46.7** [43.1, 50.3] | **321.7** [321, 322.3] | [999***, 964.0] | |
|  | **BDQ 0.03µg/ml** | **1.6** [1.7, 1.6] | **4.1** [4.4, 3.9] | **13.3** [13, 13.6] | **37.6** [40.7, 34.4] | |
|  | **BDQ 0.015µg/ml** | **0.6** [0.7, 0.6] | **0.9** [1.1, 0.7] | **2** [2.3, 1.6] | **3.6** [4.6, 2.7] | |
|  | **BDQ 0.0075µg/ml** | **0.5** [0.5, 0.5] | **0.5** [0.5, 0.5] | **0.8** [0.8, 0.7] | **1.3** [1.3, 1.2] | |
|  | **BDQ 0.00375µg/ml** | **0.4** [0.4, 0.5] | **0.4** [0.4, 0.4] | **0.7** [0.7, 0.7] | **1.1** [1, 1.1] | |
|  | **CFZ 0.06µg/ml** | **4.8** [4.4, 5.1] | **139.4** [139.4, 139.4] | **975** [976, 974] | 999*** | |
|  | **CFZ 0.03µg/ml** | **1** [0.9, 1.2] | **5.2** [5.3, 5.2] | **37.8** [40.9, 34.6] | **200**.5 [240.8, 160.3] | |
|  | **CFZ 0.015µg/ml** | **0.6** [0.6, 0.7] | **0.9** [0.9, 0.9] | **2.6** [2.8, 2.5] | **6.7** [7.1, 6.3] | |
|  | **CFZ 0.0075µg/ml** | **0.5** [0.5, 0.6] | **0.6** [0.6, 0.5] | **1.1** [1.3, 1] | **2.2** [2.5, 1.9] | |
|  | **CFZ 0.00375µg/ml** | **0.5** [0.5, 0.5] | **0.5** [0.5, 0.5] | **0.9** [1, 0.7] | **1.5** [1.7, 1.3] | |
|  | **no antib (DMSO)** | **0.5** [0.5, 0.5, 0.5] | **0.5** [0.5, 0.5, 0.5] | **0.9** [0.9, 0.8, 0.9] | **1.3** [1.3, 1.2, 1.3] | |
| **mt-DsRed2: wt-GFP** | **BDQ 0.06µg/ml** | **65.5** [69.9, 61] | **494.5** [494.5, 494.5] | *no value* | **985** [985, 985] | |
|  | **BDQ 0.03µg/ml** | **9.4** [9.5, 9.4] | **23.6** [23.3, 23.9] | **58.1** [59.9, 56.4] | **130.3** [121.6, 139] | |
|  | **BDQ 0.015µg/ml** | **3** [3, 3] | **3.1** [3, 3.2] | **3.5** [3.4, 3.6] | **3.7** [3.7, 3.7] | |
|  | **BDQ 0.0075µg/ml** | **1.5** [1.5, 1.5] | **1.2** [1.2, 1.2] | **0.9** [1, 0.9] | **0.8** [0.8, 0.8] | |
|  | **BDQ 0.00375µg/ml** | **1.3** [1.4, 1.3] | **1** [1, 1] | **0.7** [0.8, 0.7] | **0.6** [0.6, 0.6] | |
|  | **CFZ 0.06µg/ml** | **25.9** [27.3, 24.4] | **992.5** [992, 993] | 999*** | 999*** | |
|  | **CFZ 0.03µg/ml** | **5** [4.8, 5.2] | **29.5** [26.2, 32.8] | **125.2** [162.3, 88] | **728.3** [970, 486.5] | |
|  | **CFZ 0.015µg/ml** | **2.1** [2.1, 2.2] | **2.8** [2.9, 2.7] | **4.7** [4.7, 4.6] | **7.6** [7.5, 7.6] | |
|  | **CFZ 0.0075µg/ml** | **1.6** [1.6, 1.6] | **1.5** [1.5, 1.4] | **1.4** [1.4, 1.5] | **1.5** [1.5, 1.6] | |
|  | **CFZ 0.00375µg/ml** | **1.5** [1.6, 1.5] | **1.4** [1.4, 1.4] | **1.2** [1.3, 1.2] | **1.1** [1.1, 1.1] | |
|  | **no antib (DMSO)** | **1.5** [1.5, 1.5, 1.5] | **1.2** [1.3, 1.2, 1.2] | **1** [1, 1, 1] | **0.8** [0.8, 0.9, 0.8] | |

**Table S2**. **Mean values and individual replicates of the ratios of mutant vs wild type for all the concentrations of BDQ and CFZ tested, over 4 weeks**. The *M. tuberculosis* BDQ-resistant mutant, CV37 (mt), and the wild type strain, H37Rv (wt), were pooled and passaged weekly for a total of 4 weeks, in presence of subinhibitory concentrations of BDQ and CFZ. Two sets of strains were tested: mt-GFP and wt-DsRed2, and mt-DsRed2 and wt-GFP. Fluorescent-marked bacteria were counted by flow cytometry, and the ratios of the percentage of mt and wt were calculated per point. Each condition was performed in duplicate, except for the cultures containing no antibiotic, that was done in triplicate. Mean values are in bold, individual values are between brackets. *For some points at the higher BDQ and CFZ concentrations there was no wt detected (<0.1%), these cases are indicated as “999.”


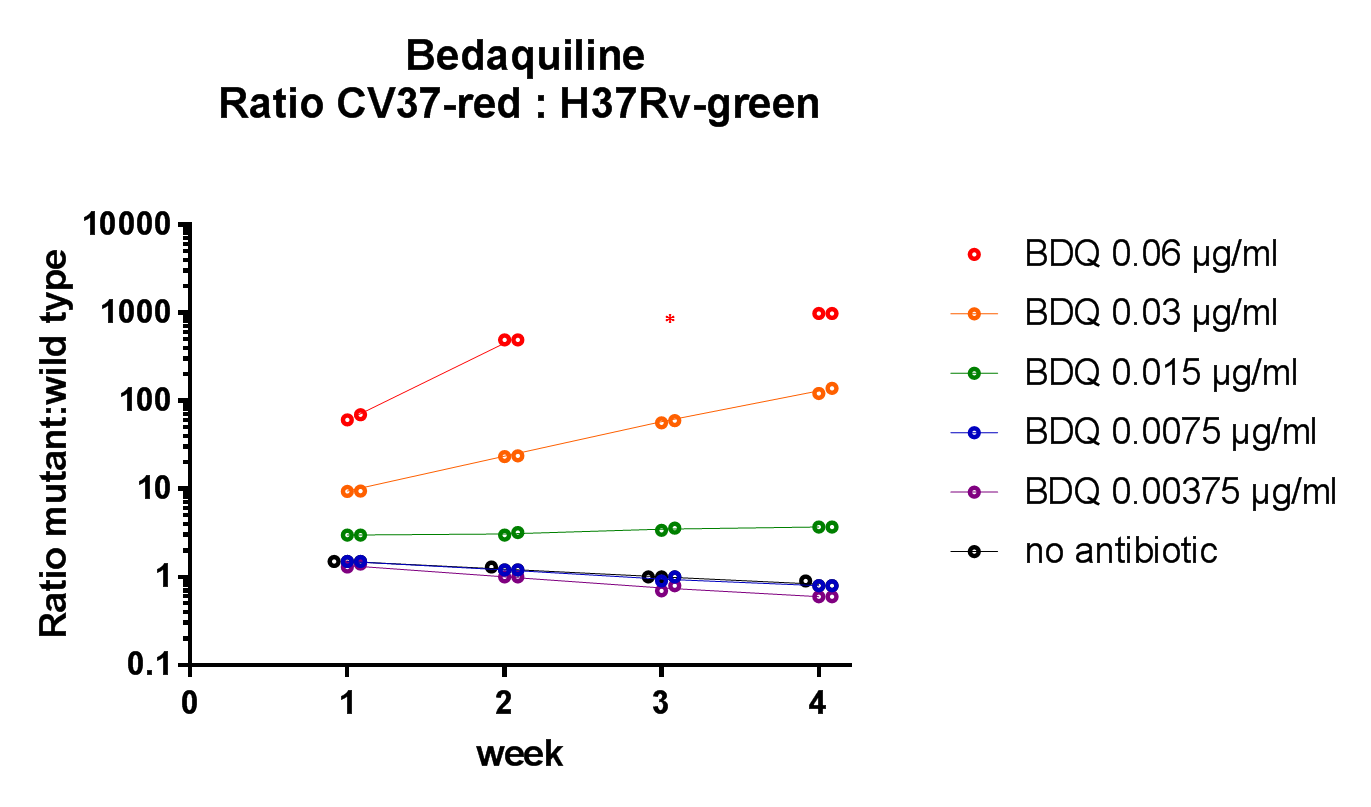

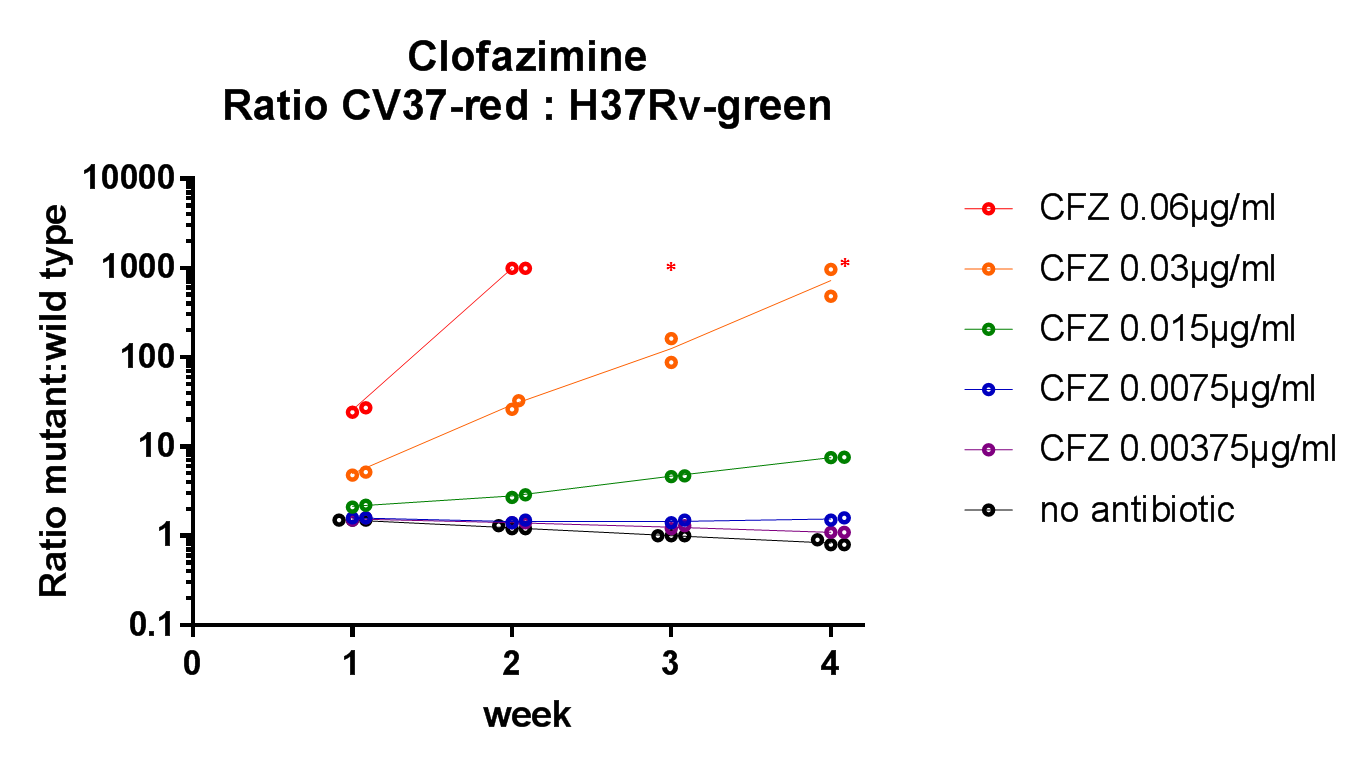


**A**

**B**

**Bedaquiline**

**Ratio mt-DsRed2 : wt-GFP**

**Clofazimine**

**Ratio mt-DsRed2 : wt-GFP**

**Fig S2**. **Ratios of** **mutant vs wild type for all the concentrations of BDQ and CFZ tested, over 4 weeks**. The *M. tuberculosis* BDQ-resistant mutant, CV37 (mt-DsRed2), and the wild type strain, H37Rv (wt-GFP), were pooled and passaged weekly for a total of 4 weeks, in presence of subinhibitory concentrations of BDQ (A) and CFZ (B). Fluorescent-marked bacteria were counted by flow cytometry, and the ratios of the percentage of mt and wt were calculated per point. Each condition was performed in duplicate, except for the cultures containing no antibiotic, that was done in triplicate. Individual values are in plotted. *For some points of BDQ and CFZ at the highest concentrations there was no wt detected (<0.1%) at weeks 3 and 4.

**Plasmid fitness**

**GFP:DsRed2**

wt-GFP: wt-DsRed2

mt-GFP: mt-DsRed2

**Fig S3**. **Fitness of GFP vs DsRed2 strains.** H37Rv (wt)-GFP vs wt-DsRed2, were pooled and passaged weekly for a total of 4 weeks, without antibiotic. The same procedure was done with the mutant CV37 strains (mt-GFP and mt-DsRed2). Fluorescent-marked bacteria were counted by flow cytometry, and the ratios of the percentage of mt and wt were calculated per point. Each condition was performed in duplicate. Individual values are in plotted.
